# Supplementary material for: Genomic analysis of focal nodular hyperplasia with associated hepatocellular carcinoma unveils its malignant potential: a case report
Source: Commun Med (Lond). 2022 Feb 3;2:11. doi: 10.1038/s43856-022-00074-y (PMC9053256; doi:10.1038/s43856-022-00074-y)
Supplement: Supplementary file 1 — Supplementary Information [file 43856_2022_74_MOESM1_ESM.pdf]

Genomic Analysis of Focal Nodular Hyperplasia with Associated Hepatocellular Carcinoma Unveils its Malignant Potential

Caner Ercan[1,2*], Mairene Coto-Llerena[1,2*], John Gallon[1*], Lana Fourie[1,3], Mattia Marinucci[1], Gabriel F. Hess[1,3], Jürg Vosbeck[2], Stephanie Taha-Mehlitz[3], Tuyana Boldanova[3,4], Marie-Anne Meier[4], Alexandar Tzankov[2], Matthias S. Matter[2], Martin H. K. Hoffmann[5], Luca Di Tommaso[6,7], Markus von Flüe[3], Charlotte K. Y. Ng[8,9], Markus H. Heim[3,4], Savas D. Soysal[3], Luigi M. Terracciano[6,7], Otto Kollmar[3] and Salvatore Piscuoglio[1,2]

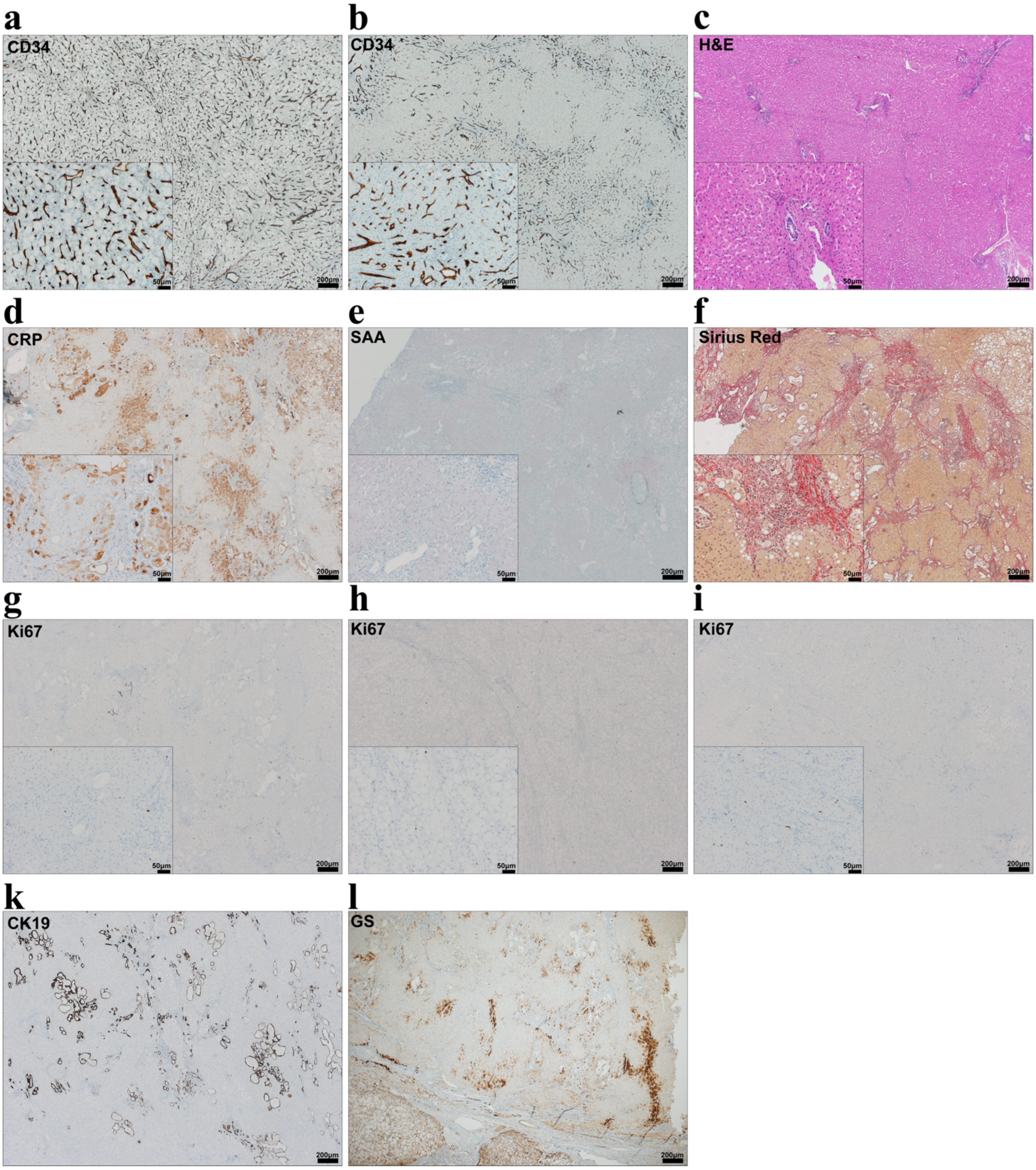

Fig. S1: Histopathological characterization of the Focal Nodular Hyperplasia (FNH) and Hepatocellular Carcinoma (HCC). (a) In Hepatocellular Carcinoma 1 (HCC1), CD34 staining revealed diffuse capillarization of sinusoids. (scale bar 200 µm; insert 50 µm) **(b)** Hepatocellular Carcinoma 2/ High-grade dysplastic nodule HCC2/HGDN has capillarization of sinusoids in most of the areas by CD34 immunohistochemistry (IHC). (scale bar 200 µm; insert 50 µm) **(c)** The background liver has no pathology. (scale bar 200 µm; insert 50 µm) **(d)** The Focal Nodular Hyperplasia (FNH) shows patch positivity for c-reactive protein (CRP). While the HCC1 has diffuse positivity (scale bar 200 µm; insert 50 µm). **(e)** Serum Amyloid A IHC was negative on FNH and both HCC1 and HCC2/HGDN. (scale bar 200 µm; insert 50 µm) **(f)** The FNH was intersected by many bile duct-rich fibrotic bands (Sirius Red). (scale bar 200 µm; insert 50 µm) **(g-i)** The Ki-67 proliferative index was lower than 1% for FNH **(g)**, HCC1 **(h)**, HCC2/HGDN**(i)**. **(k)** CK19 positive bile ducts in fibrotic bands of FNH. (scale bar 200 µm; insert 50 µm) **(l)** GS was “map-like” positive on FNH. (scale bar 200 µm)

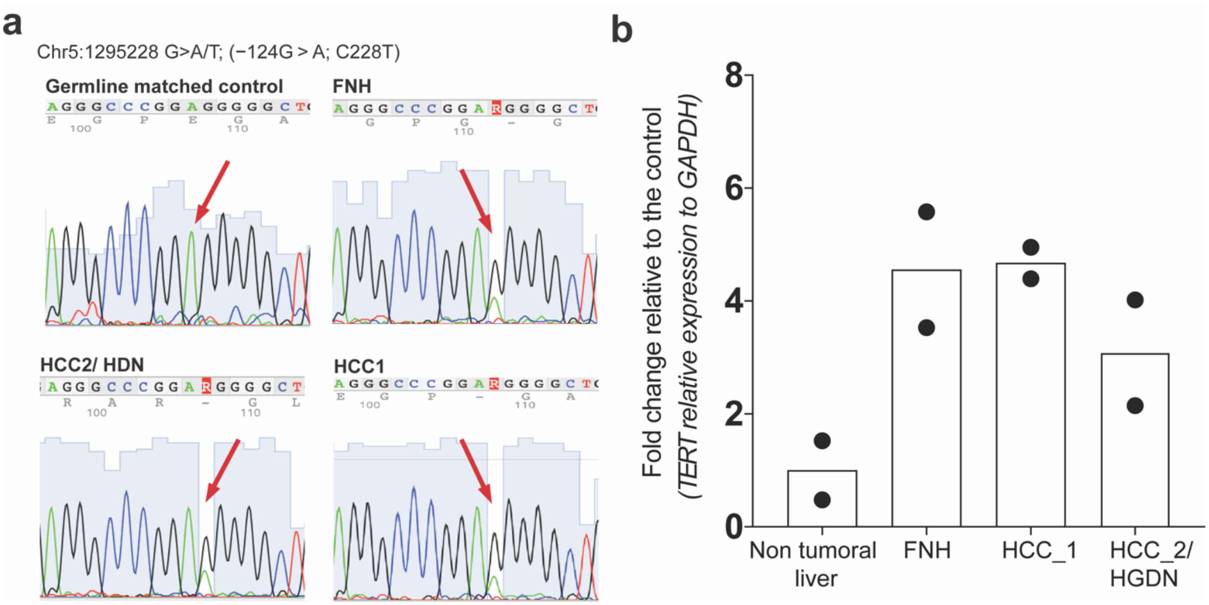

Fig. S2: *TERT* promoter status in the Focal Nodular Hyperplasia (FNH) and Hepatocellular Carcinoma (HCC). (a) Representative sequence electropherograms (tumor and matched germline) of the samples subjected to Sanger sequencing for the analysis of the *TERT* promoter. **(b)** *TERT* mRNA levels in the lesions harbouring the TERT promoter hotspot mutations as assessed by quantitative real-time PCR (qRT-PCR).
