# Supplementary material for: Genomic analysis of focal nodular hyperplasia with associated hepatocellular carcinoma unveils its malignant potential: a case report
Source: Commun Med (Lond). 2022 Feb 3;2:11. doi: 10.1038/s43856-022-00074-y (PMC9053256; doi:10.1038/s43856-022-00074-y)
Supplement: Supplementary file 5 — Description of Additional Supplementary Files [file 43856_2022_74_MOESM5_ESM.pdf]

Description of Additional Supplementary Files

File Name: Supplementary Data 1

Description: List of somatic mutations identified in the FNH and the two HCC components

File Name: Supplementary Data 2

Description: The source data underlying the graphs and charts presented in Fig S2b
